# Supplementary material for: miRNA-mediated TUSC3 deficiency enhances UPR and ERAD to promote metastatic potential of NSCLC
Source: Nat Commun. 2018 Nov 30;9:5110. doi: 10.1038/s41467-018-07561-8 (PMC6269493; doi:10.1038/s41467-018-07561-8)
Supplement: Supplementary file 1 — Supplementary Information [file 41467_2018_7561_MOESM1_ESM.pdf]

SUPPLEMENTARY INFORMATION

MiRNA-mediated TUSC3 deficiency enhances UPR and ERAD to promote metastatic potential of NSCLC.

Jeon et al.,

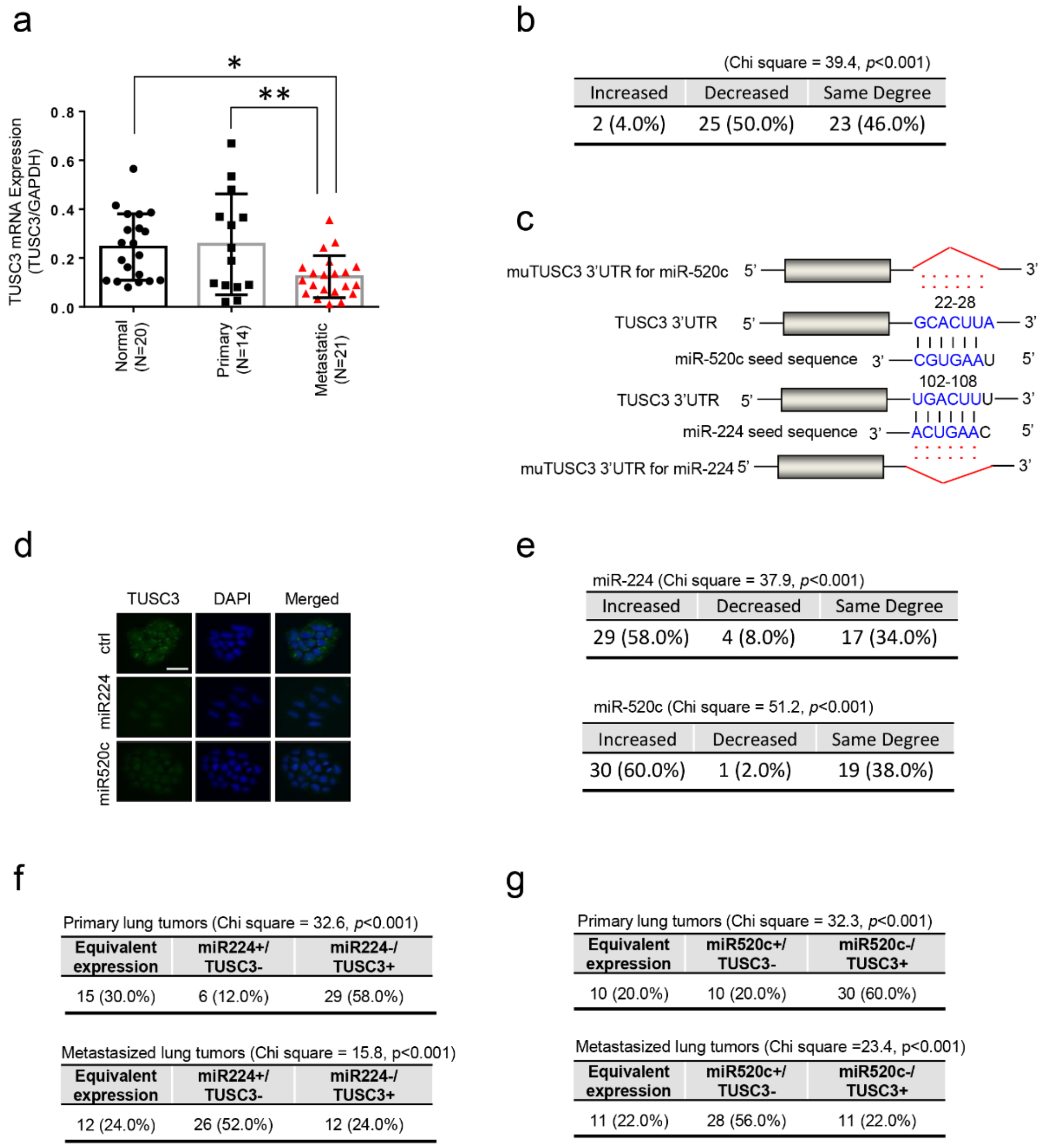

Supplementary Figure 1. TUSC3 was suppressed by miR-224 and -520c in metastasized lung tumors.

a, qRT-PCR analysis showing the mRNA expression of TUSC3 in normal, primary, or metastatic lung tumor samples. The RNA samples were purified from 20 normal, 14 primary or 21 metastatic lung tissue samples and subject to qRT-PCR with TUSC3 or GAPDH probe. Bars indicated means ±SD and *p*-values were obtained by unpaired student t-test (**p*= 0.0014, ** *p*=0.0127) **b**, Summary table of immunohistochemistry (IHC) showing the number of cases differentially expressed in metastasized lung cancers compared to the primary tumors. **c**, Schematic diagram showing complementarity between seed sequences on miR-224/-520c and

the 3'UTRs of TUSC3. **d**, Immunofluorescence (IF) assay showing the decreased expression of TUSC3 in miR-224 or miR-520c overexpressing H460 cells. MiR-224 or miR-520c was ectopically expressed in H460 cells. After 48 h, the cells were prepared for immunostaining with anti-TUSC3 antibody. The scale bar on the image indicates 100 μm **e**, The enhanced expression of miR-224 or 520C in metastatic lung cancers compared to the primary tumors. **f,g**, The summarized tables showing significant inverse correlation between TUSC3 and miR-224/-520c expression in lung cancer patient samples. Equivalent expression indicates that either a given core did not express either TUSC3 or the miRNAs or that a given core expressed both targets. In the latter case, co-expression analysis showed that the cancer cells expressing TUSC3 were always distinct from those expressing the miRNAs.

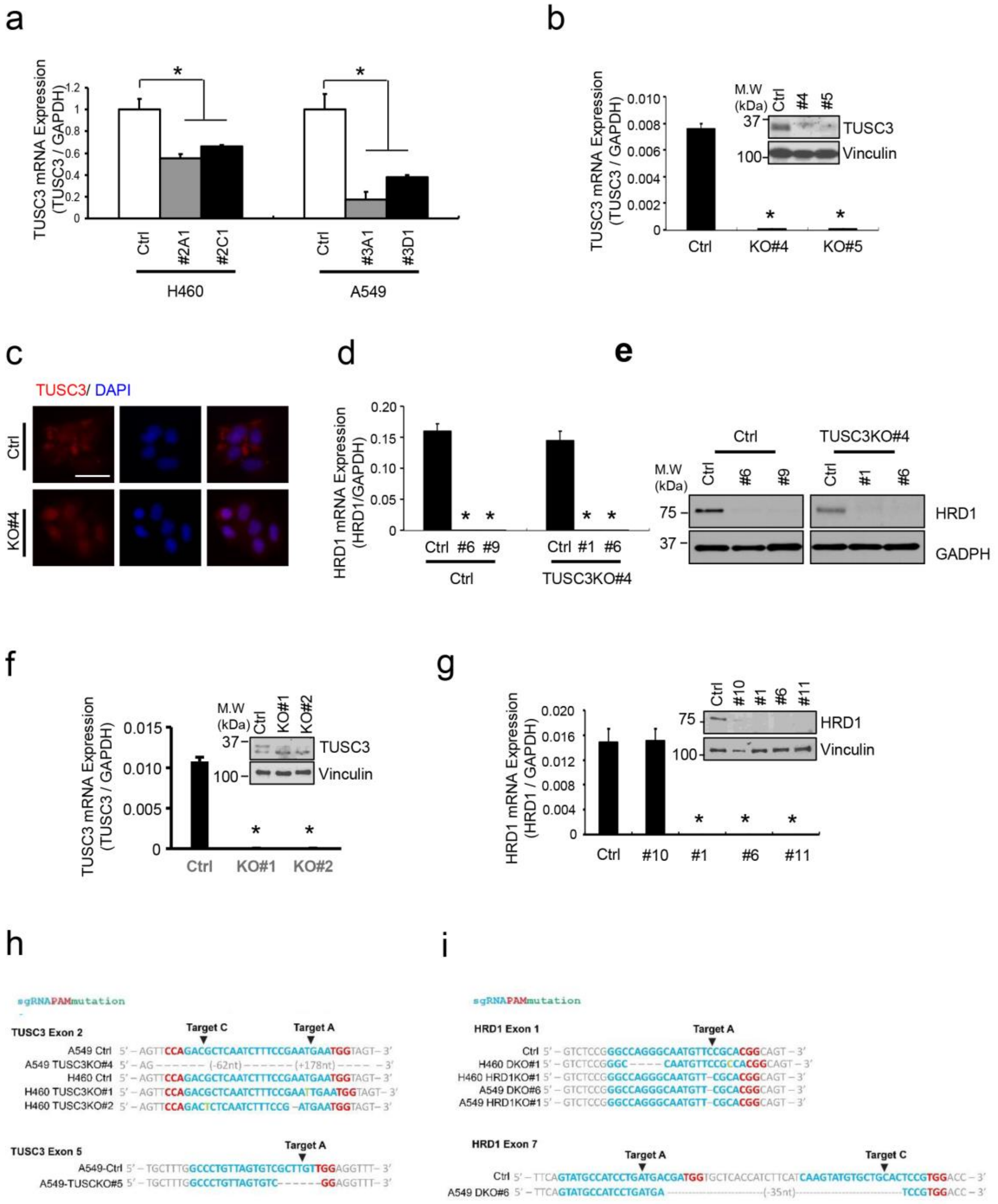

Supplementary Figure 2. The establishment of TUSC3 deficient cells using TUSC3 shRNAs or CRISPR TUSC3 knock-out (KO) constructs. a, qRT-PCR analysis validating decreased TUSC3 mRNA expression in A549 and H460 TUSC3 KD candidates. Bars represent means ±SD (n=3) and the *p*-values were determined by two-tailed student t-test (**p*<0.05). **b,c,** Validation of TUSC3 KO generation using qRT-PCR (b, bottom panel), Western blot analysis (b,

upper panel) or IF assay (c) in A549 TUSC3KO cells. The scale bar in (c) showed 100 μm **d,e**, Validation of HRD1 KO cells or TUSC3/HRD1 KO cells. The A549 HRD1/TUSC3 DKO cells were generated from A549 TUSC3KO#4 cells using CRISPR HRD1 constructs as described in Methods. The generation of the KO cells was confirmed by qRT-PCR **(d)** or Western bot **(e)** analyses. Bars indicate means ±SD (n=3) and the *p*-values were determined by two-tailed student t-test (*p<0.005). **f,g**, The generation of H460 TUSC3KO or H460 HRD1KO cells using CRISPR KO constructs, respectively. The gene expression was validated by qRT-PCRs and Western blot analyses. The bars indicate means ±SD (n=4) and the *p*-values were determined by two-tailed student t-test (*p<0.005). h,i, The validation of TUSC3 or TUSC3/HRD1 DKO cells by genomic DNA sequences. The information about the sgRNAs and lists of primers are shown in Supplementary Table 7.

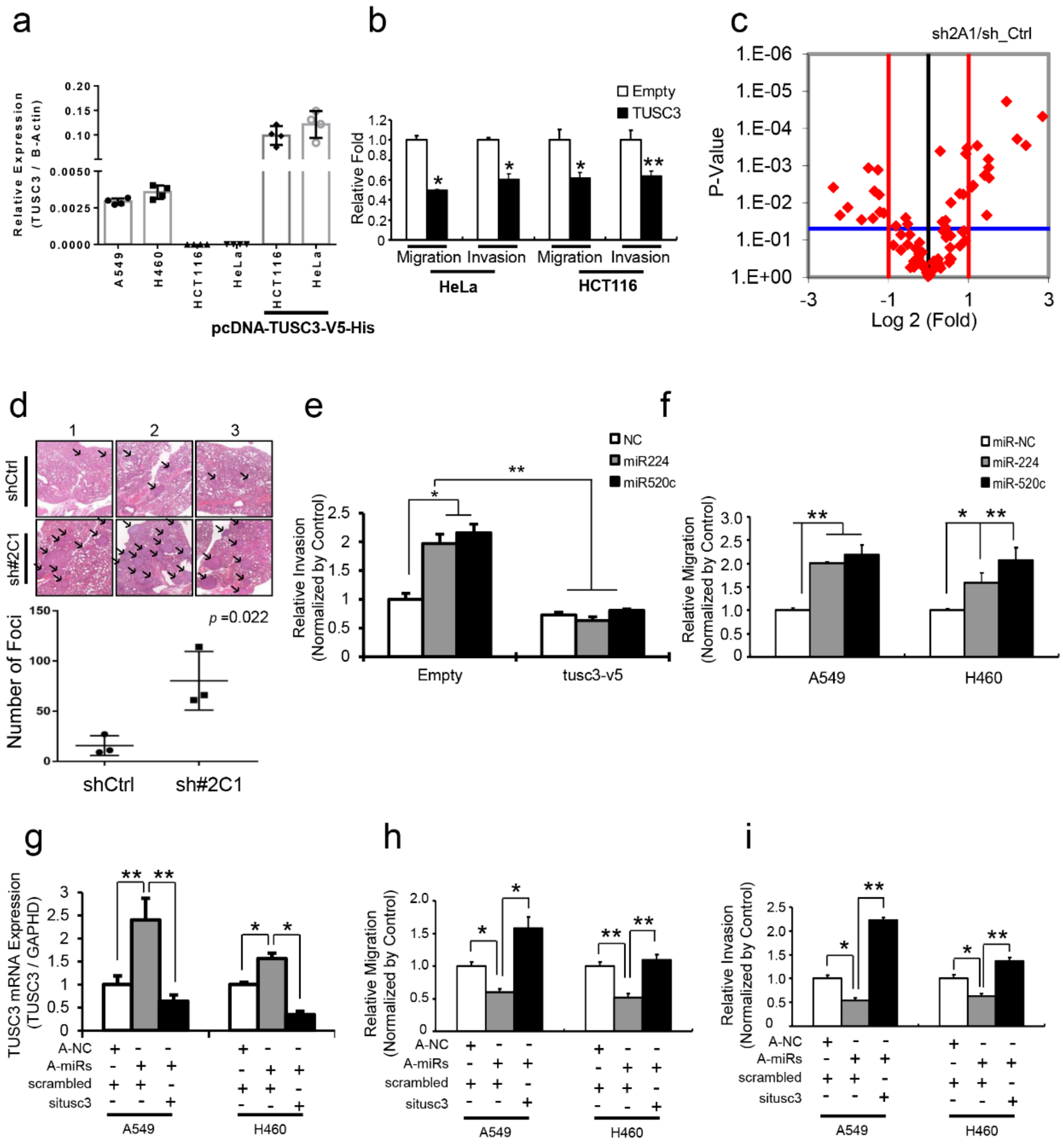

Supplementary Figure 3. MiR-224/-520c-dependent TUSC3 suppression enhances metastatic potential of NSCLC *in vitro* and *in vivo*. a,b Suppressed migratory and invasive abilities in TUSC3 null cells by TUSC3 overexpression. The HCT116 and HeLa cells were transfected by either empty or pcDNA-TUSC3-v5-his vectors for 24 h, and the cells were prepared for qRT-PCR reactions (a) or subject to migration and invasion chambers for 20-40 h (b), respectively. After that, the migratory or invasive cells were detached using detaching solution and quantified by Calcein AM staining. Bars indicate means ±SD (n=4 for qRT-PCR, n=3 for migration/invasion assays) and the *p*-values were calculated by two-tailed student t-test (*p < 0.005, **p < 0.02). **c,** Volcano plots showing differential expressions of 84 known

metastasis-related genes in H460 TUSC3KD cells (sh#2A1). The lists of the candidates are shown in Supplementary Table 1. **d,** Increased tumor burden in orthotopic xenograft mice model with H460 TUSC3 KD cells. $5x10^5$ H460 control (shCtrl) or H460 TUSC3 KD cells (sh#2C1) were intravenously injected into three nude mice of each group. After 4 weeks, the lung tissues were obtained and prepared for H&E staining (upper panel) and the numbers of foci were counted under light microscope (lower panel). Bars represent means ±SD (n=3) and *p*-values were calculated by two-tailed student t-test. **e, f,** The enhanced migration and invasion of the lung cancer cells overexpressing miR-224 or miR-520c. The H460 or A549 cells were transfected with pre-miR-224 or pre-miR-520c and/or pcDNA-TUSC3-V5-His vector for 48 h. The cells were subsequently subject to invasion or migration assay. After 20-40 h, the invasive and migratory cells were quantified by MTS assay. Bars indicate means ±SD (n=4) and *p*-values were calculated by student t-test (**p*<0.005 and ***p*<0.001). **g-i,** miR-224/-520c-dependent TUSC3 suppression is responsible for enhanced migratory and invasive abilities of the lung cancer cells. Anti-miR-224/-520c inhibitors were transfected with scrambled or siTUSC3 siRNAs in A549 or H460. After 48 h, the cells were harvested and prepared for Western blot or qRT-PCR analyses to confirm the altered expression of TUSC3 by the anti-miRNAs and/or siTUSC3 siRNAs **(g)**. The remaining cells were subject to analyzing the capacities of the anchorage independent cell growth (Fig. 2e), migration **(h)** or invasion **(i)**. Bars indicate means ±SD (n=4) and *p*-value were calculated by student t-test (**p*<0.005 and ***p*<0.01).

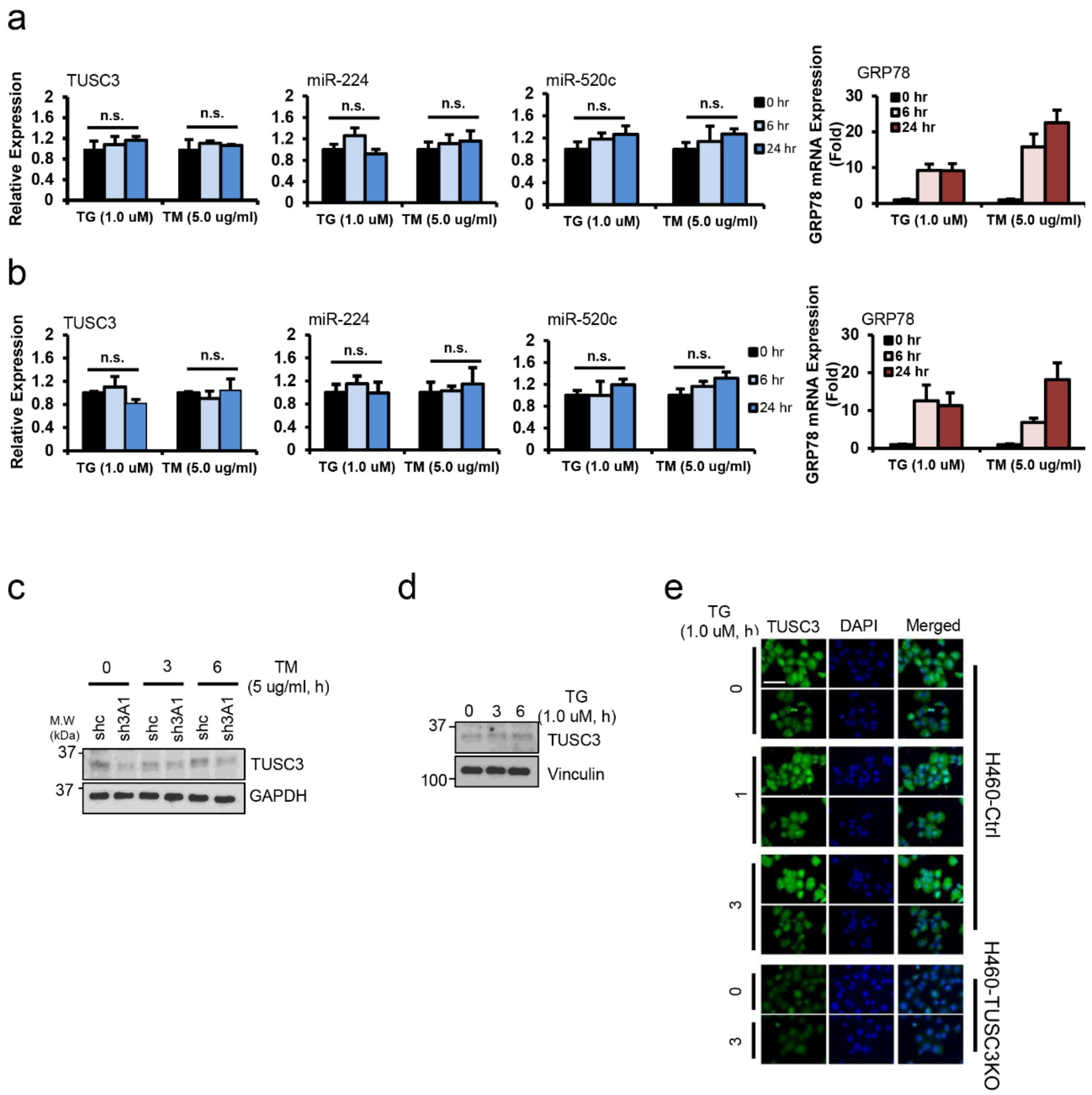

Supplementary Figure 4 Unchanged expression of TUSC3 in response to ER stress induction in lung cancer cells. a-d, Steady state expression of TUSC3, miR-224 and miR-520c upon ER stress induction. An ER-stress inducible drug, Thapsigargin (TG) or Tunicamycin (TM) was treated into H460 **(a)** or A549 **(b)** cells as indicated. Subsequent qRT-PCR **(a,b)** or Western blot **(c,d)** analyses showing unchanged mRNA and protein levels of TUSC3 in response to ER stress induction. **e,** Immunofluorescence analysis showing unchanged location of TUSC3 protein in response to ER stress induction. The H460 cells were incubated with TG as indicated. Subsequently, the TUSC3 protein was analyzed by IF assay using an anti-TUSC3 antibody. The IF stain in H460 TUSC3 KO cells showed the specificity of the anti-TUSC3 antibody. The scale bar indicates 100 μm.

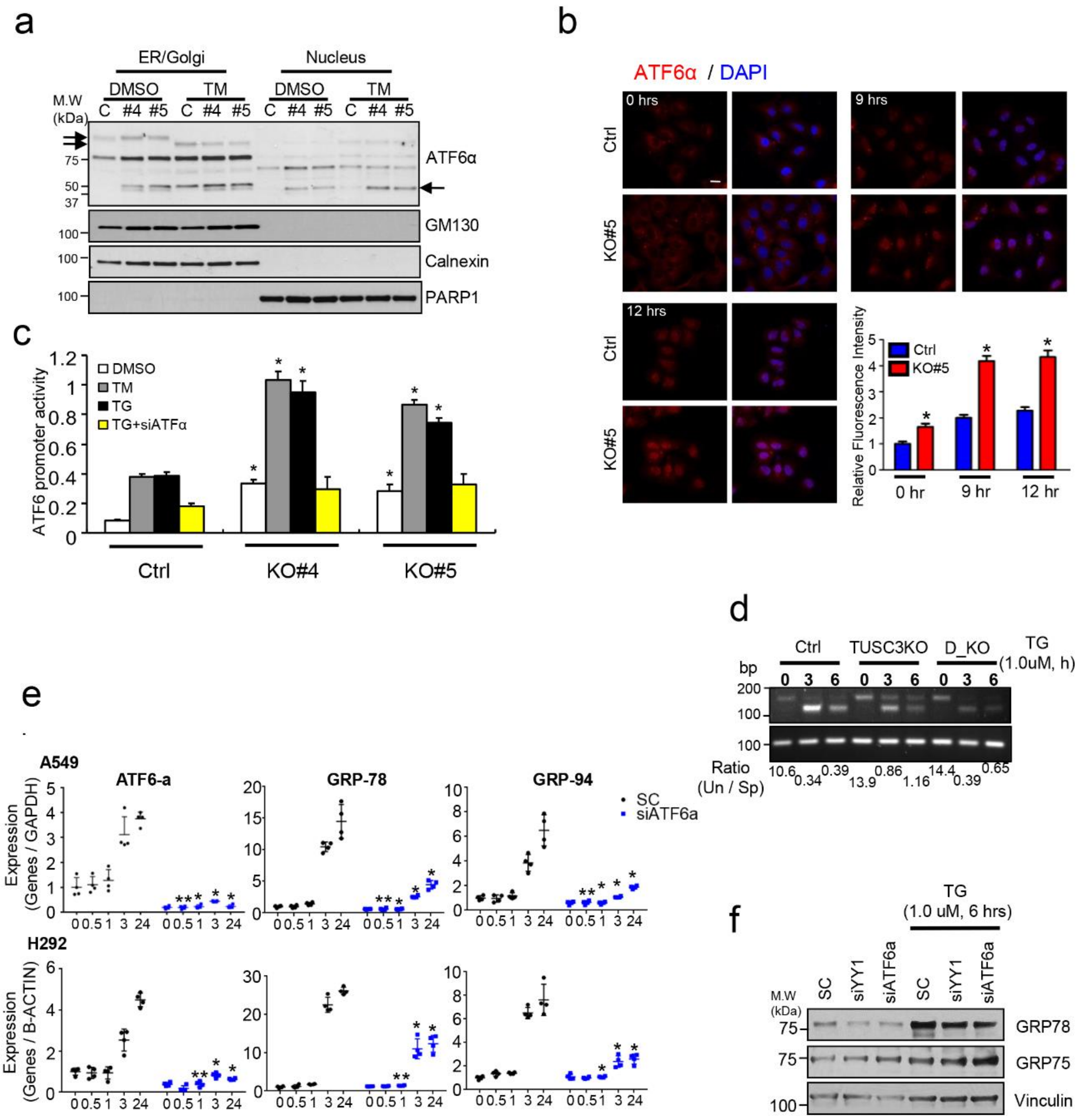

Supplementary Figure 5 The hyperactivation of ATF6α pathway in TUSC3 deficient cells. **a**, Enhanced nuclear localization of active ATF6α in A549 TUSC3KO cells. Subcellular fractionation assay was performed using A549 control or TUSC3KO cells (#4 and #5) treated with TM (3ug/ml) for 9h. The arrows from the top indicate glycosylated, unglycosylated or active ATF6α proteins, respectively. Anti-Calnexin, anti-GM130 and anti-PARP-1 antibodies were used for ER, Golgi and Nuclear fraction markers, respectively. **b**, Immunofluorescence assays with anti-ATF6α antibody showing increased nuclear-localized ATF6α protein in A549 TUSC3 KO cells (KO#5). The indicated cells were incubated with TM (3 ug/ml) for 9 or 12 h

and the cells were stained by anti-ATF6α antibody. Th expression pattern was analyzed and quantified by Zeiss 780 Confocal microscope. The summarized result is shown at right bottom. Bars represent means ±S.E.M (n=20) and *p*-values were obtained by two-tailed student t-test (**p*<0.001). **c,** Upregulated ATF6α promoter activities in A549 TUSC3KO cells (KO#4 and KO#5). The luciferase constructs containing ATF6α promoter elements were co-transfected with pcDNA-Renilla plasmid and/or siATF6α siRNAs for 48 h. The cells were subsequently treated by TM (1 ug/ml) or TG (0.5 uM) for 24 h. The promoter activities were calculated by normalizing firefly promoter values by Renilla values, respectively. Bars indicate means ±SD (n=6) and *p*-value was obtained by paired student t-test (**p*<0.0001) and size bar on image shows 100 μm. **d,** Alteration of XBP1 mRNA in A549TUSC3 KO and HRD1/TUSC3 DKO cells. The cells were stimulated by TG as shown in the figure and prepared for RNA purification. Subsequently, the samples were subject to reverse transcription PCR analysis using GAPDH and XBP1 primers for 25 cycles. The band intensity was measured by Image J software and the values were shown by ratio between unspliced (Un) and spliced (Sp) followed by normalization with that of GAPDH. *The information about primer sequences for XPB1 cleavage was obtained from previous literature[1]. **e,** ATF6α-dependent expression of ER heat shock proteins. siATF6α siRNAs were transiently treated into A549 and H292 cells for 48 h and subsequently qRT-PCR analysis was performed with GRP78 and GRP94 probes. Bars represent means ±SD (n=4) and *p*-value were obtained by paired student t-test (**p*<0.01, ** *p*<0.05). **f,** ATF6α-dependent expression of GRP78, but not a mitochondrial heat shock protein, GRP75 protein. After knocking-down of ATF6α or YY1 by corresponding siRNAs, Western blot analysis was performed using indicated antibodies.

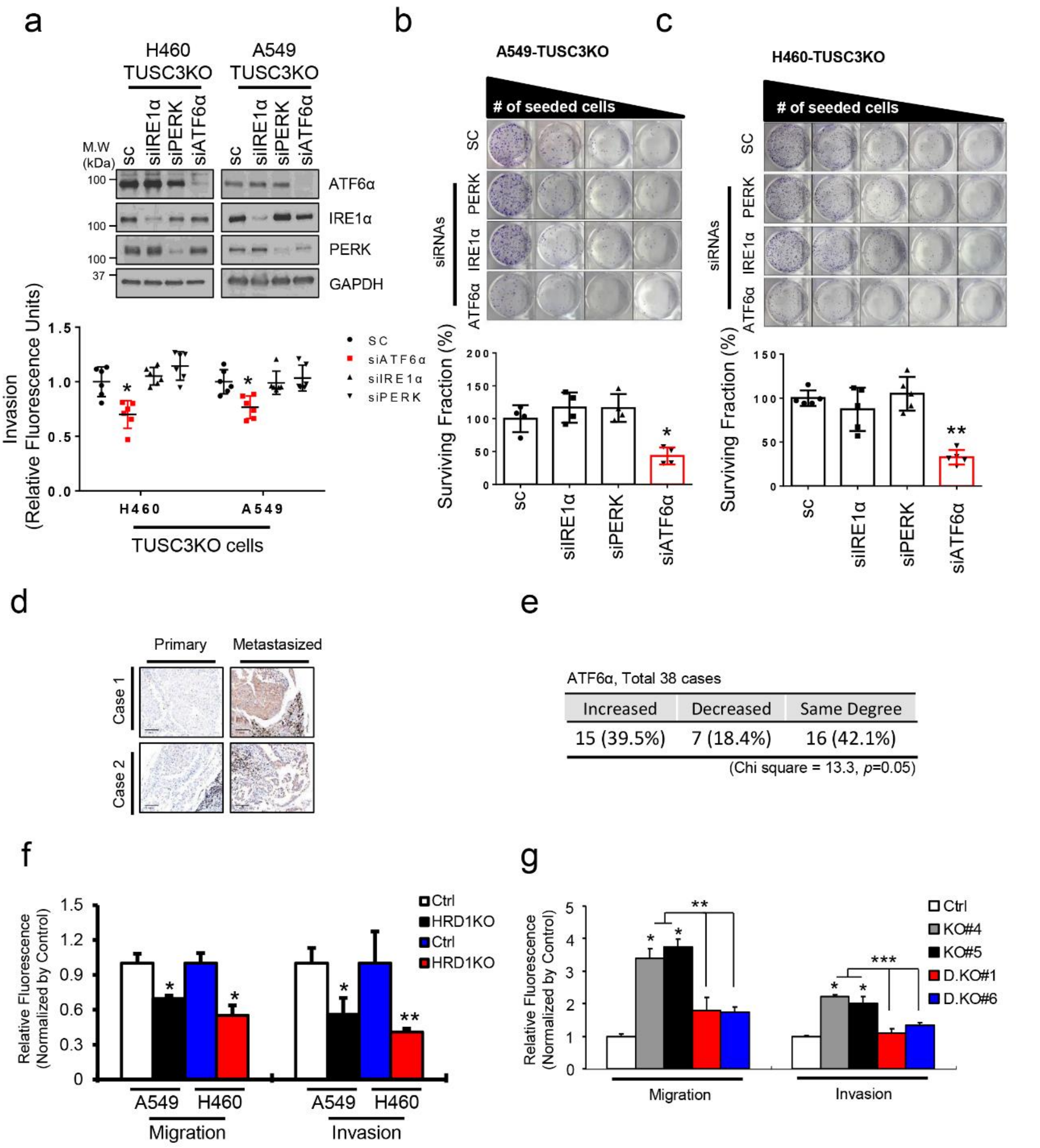

Supplementary Figure 6 The metastatic potential of TUSC3 deficiency is rescued by ATF6α or HRD1 downmodulation. a, Decreased invasive ability of TUSC3KO cells by suppressing ATF6α but not IRE1α or PERK expression. The KO cells were transiently transfected siIRE1α, siPERK or siATF6α siRNAs. After 24 h, the cells were subject to invasion chamber and incubated for additional 40 h. The invaded cells were quantified by Calcein AM staining. The KD efficiency for each siRNAs were addressed by Western blot analysis with indicated antibodies. Bars indicate means ±SD (n=6) and the *p*-values were calculated by two-

tailed student t-test. (*p < 0.005). **b,c,** The suppression of ATF6α reduced clonogenicity of A549 **(b)** or H460 **(c)** TUSC3KO cells. The IRE1α, PERK or ATF6α was transiently knock downed by their corresponding siRNAs and subsequently spread into 6 well plate by serial dilution. After 10-14 days, the clonogenic effects were addressed by calculating survival fraction of each KD cells and normalized by that of control KO cells transfected by scrambled RNAs (SC). Bars indicate means ±SD and the p-values were calculated by two-tailed student t-test. (**b,** N=4 *p < 0.01; **c**, N=5, **p < 0.001). **d,e** IHC analysis using anti-ATF6a antibody showing that enhanced expression and/or nuclear localization of ATF6α in metastasized lung cancer patient samples compared to primary lung cancer. The cases are summarized for total 38 paired sets and p-value was calculated by chi square t-test. The quantification was done by the InForm system that can quantify the percentage of cells of a given type positive for the target of interest as previously described[2]. *Note that total 40 paired sets were applied to IHC using anti-TUSC3 and anti-ATF6α antibodies. But two cores for anti-ATF6α were accidently missing. Thus total 78 cases for co-expression analysis **(Fig. 3i)** and 38 paired sets for ATF6α **(d,e)** were subject to analyze.

f, Decreased migration and invasion by HRD1 deficiency. The indicated cells were subject to migration or invasion chamber and incubated for 20-40 h. The migratory or invasive cells were quantified using Calcein AM staining as the following manufacturer's protocol. Bars represent means ±SD (n=5) and the p-values were calculated by two-tailed student t-test. (*p < 0.001 and **p < 0.01). **g,** Re-suppressed migratory and invasive capacities in response to HRD1 downregulation in A549 TUSC3 KO cells. A549 TUSC3 KO (KO#4 or KO#5) or A549 HRD1/TUSC3 DKO (D.KO#1 or D.KO#6) cells were subject to migration or invasion chamber and incubated for 24 h (migration) and 40 h (invasion). Error bars show means ±SD (n=4) and p-values were obtained by two tailed student t-test (*p<0.001, **p<0.01 and ***p<0.02).

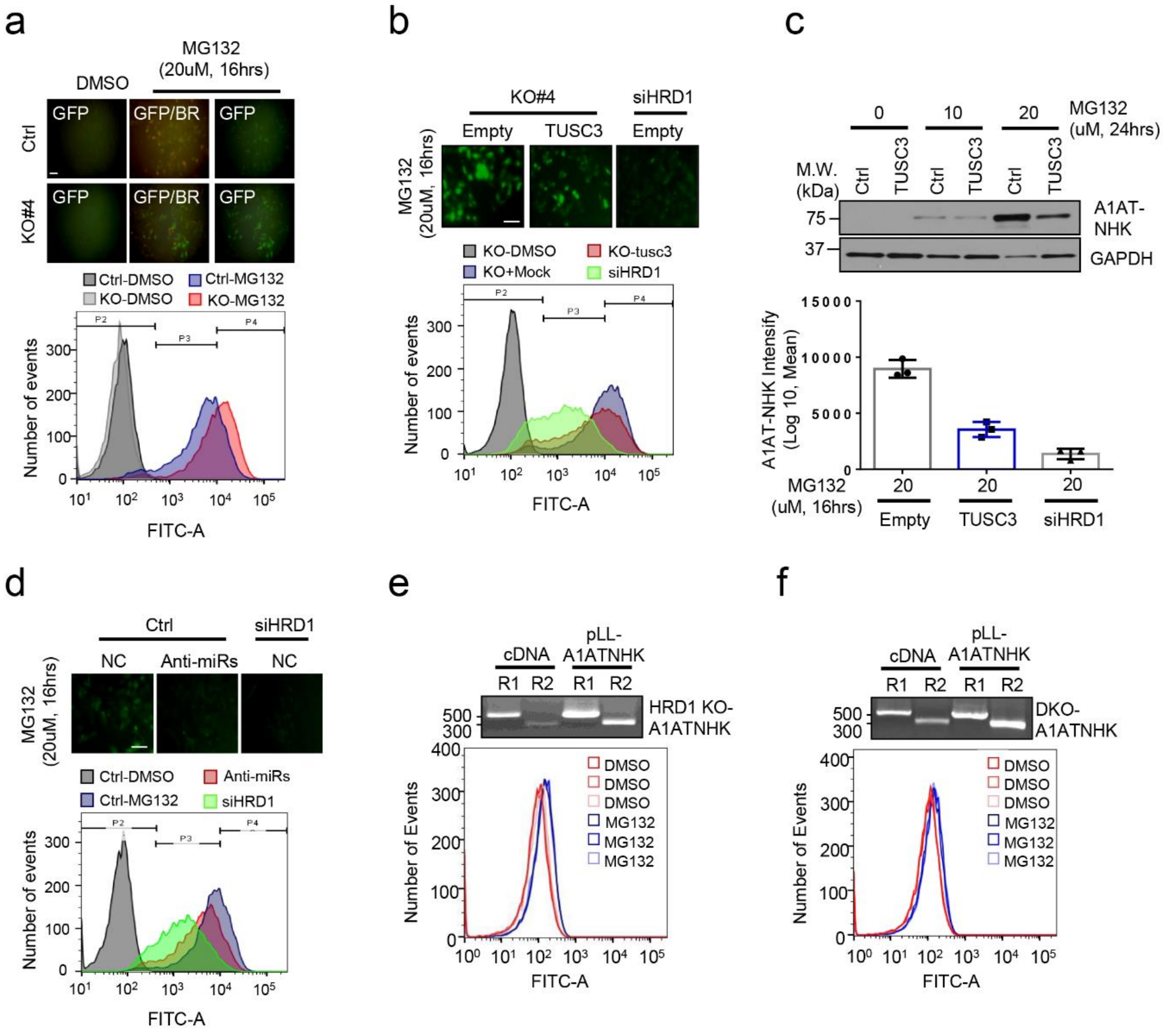

Supplementary Figure 7 Enhanced HRD1-dependent ERAD in miR-224/-520c-dependent TUSC3 suppression. a, A1AT-NHK-ddVenus, an ERAD substrate accumulation in TUSC3 KO cells. A549 or A549 TUSC3 KO stably expressing A1AT-NHK-ddVenus was incubated with 20uM of MG132 for 16 h to inhibit proteasome activity, resulting in the accumulation of A1AT-NHK-ddVenus. The protein accumulation was monitored under Fluorescence microscope (Olympus, CKX4, upper panels) and quantified by FACS analysis (bottom panels). The scale bar on image shows 20 μm **b,** Decreased A1AT-NHK-ddVenus accumulation by TUSC3 reconstitution in A549 TUSC3 KO cells. The pcDNA-TUSC3-V5-His plasmids or siHRD1 siRNAs were transiently expressed in A549 TUSC3 KO-A1AT-NHK-ddVenus cells followed by treating MG132 (20 uM) for 16 h. The protein accumulation was monitored by Zeiss Axioskop 40 microscopes with a Zeiss AxioCam HRc camera and quantified by FACS analysis (bottom

panels). **c,** Decreased A1AT-NHK-ddVenus accumulation by rescued TUSC3 gene in A549 TUSC3KO-A1AT-NHK-ddVenus cells. pcDNA-TUSC3-V5-His vector was reconstituted into the TUSC3KO cells, and the cells were incubated with MG132 for 24h and the protein accumulation was analyzed by Western blot analysis. Bottom bar graph is the summarized data showing the Mean A1AT-NHK-ddVenus intensity measured by FACS analysis. **d,** The suppression of miR-224 and -520c showed decreased A1AT-NHK-ddVenus accumulation indicating decreased HRD1-dependent ERAD efficiency. The size bars on the images of **(b)** and **(d)** indicate 100 μm. **e,f,** Unresponsiveness of A1AT-NHK-ddVenus to MG132 treatment in A549 HRD1KO cells. The A549 HRD1KO-A1AT-NHK-ddVenus (e) or TUSC3/HRD1KO (DKO)-NHK-ddVenus (f) cells were treated with MG132 (20uM) for 16 h, and the protein accumulation was subsequently monitored by FACS analysis. The mRNA expression of A1AT-NHK-ddVenus was validated by Agarose gel images for A1AT-NHK-ddVenus probes. The pLL-A1ATNHK plasmids were used for the positive controls of the PCR probes.

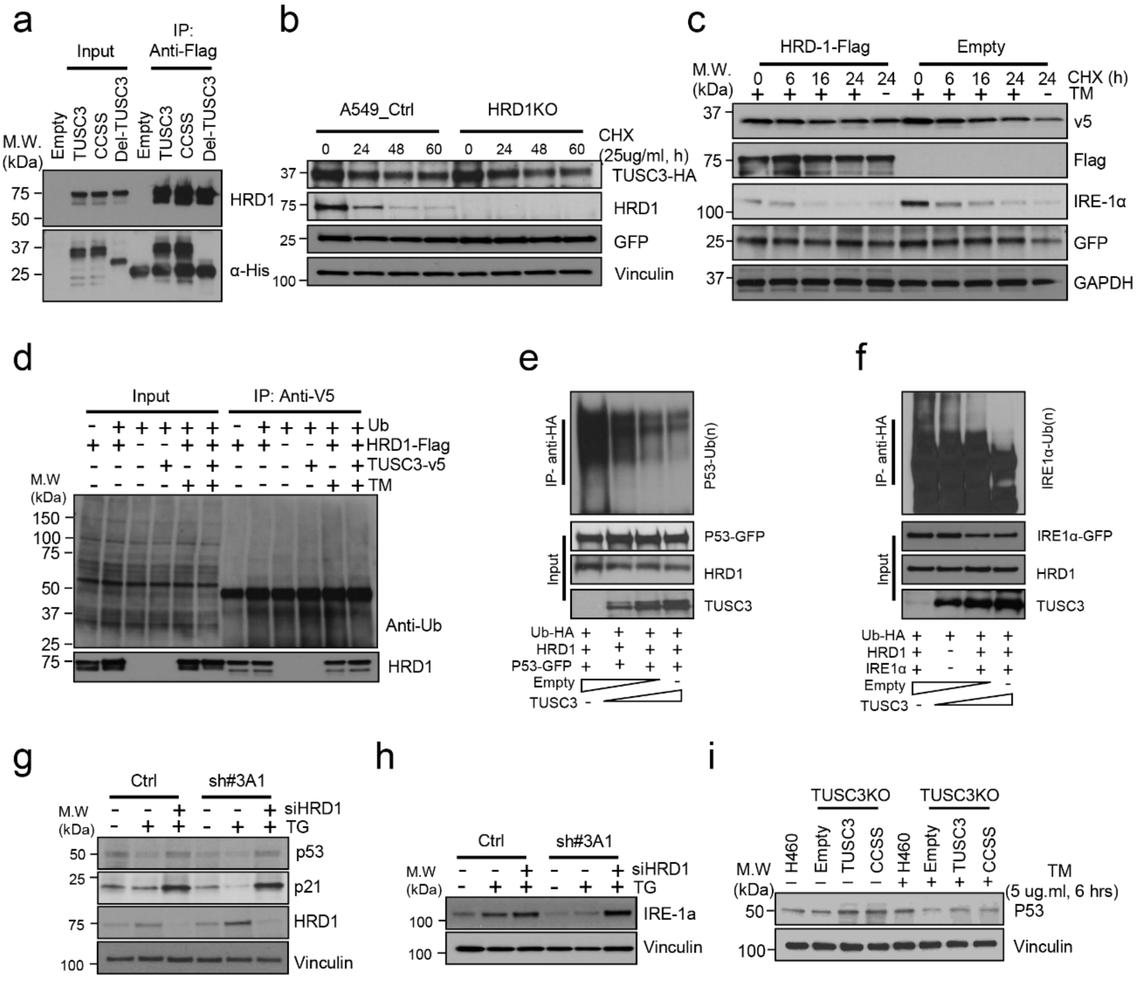

j

Primary lung tumors for p53 and TUSC3

P53+ / TUSC3+	P53+ / TUSC3-	P53- / TUSC3+	P53- / TUSC3-
11 (27.5%)	5 (12.5%)	16 (40.0%)	8 (20.0%)

(Chi square = 1.3, *p* =0.1)

Metastasized lung tumors for p53 and TUSC3

P53+ / TUSC3+	P53+ / TUSC3-	P53- / TUSC3+	P53- / TUSC3-
12 (30.0%)	4 (10.0%)	12 (30.0%)	12 (30.0%)

(Chi square = 4.0, *p* <0.05)

k

Primary lung tumors for IRE-1a and TUSC3

IRE1a+ / TUSC3+	IRE1a+ / TUSC3-	IRE1a- / TUSC3+	IRE1a- / TUSC3-
17 (42.5%)	8 (20.0%)	9 (22.5%)	6 (15.0%)

(Chi square = 28.8, *p* <0.005)

Metastasized lung tumors for IRE-1a and TUSC3

IRE1a+ / TUSC3+	IRE1a+ / TUSC3-	IRE1a- / TUSC3+	IRE1a- / TUSC3-
26 (65.0%)	8 (20.0%)	0 (0%)	6 (15.0%)

(Chi square = 36.0, *p* <0.005)

Supplementary Figure 8 Interaction between HRD1 and TUSC3 proteins regulating ERAD efficiency of the HRD1 on its substrates. a, Co-immunoprecipitation assay between TUSC3 and HRD1 protein. The pCMV6-HRD1-Flag plasmid was co-transfected with the indicated plasmids, respectively. After 24 h, the cells were lysed by 1%CHPAS buffer for 1h followed by immunoprecipitation using anti-Flag antibody. The interactions of TUSC3 or its

mutants were determined by Western blot analyses with anti-His antibody. **b,** Pulse-Chase analysis with TUSC3 protein in HRD1 KO cell. The cells were transfected with pcDNA-TUSC3-HA and pEGFPN1 for 24 h and the cells subsequently incubated with Cycloheximide (CHX, 25 ug/ml) for indicated times. The amount of the proteins was shown by Western blot analysis using indicated antibodies. The expression of the GFP protein was used for the transfection efficiency. **c,** Unchanged stability of TUSC3 protein in A549 TUSC3/HRD1 KO cells. pcDNA-TUSC3-V5-His and pEGFPN1 were co-transfected with either empty or pCMV6-HRD1-Flag. After 24 h, the cells were incubated with TM (5 ug/ml) for 6 h and subsequently CHX (50 ug/ml) was treated for indicated times. **d,** *In vivo* ubiquitination assay of TUSC3-V5-His protein in HRD1 overexpressing cells. HEK293 cells were transfected with indicated plasmids for 24 h and subsequently exposed to TM (3 ug/ml) and MG132 (8 uM) for 6 h followed by immunoprecipitation reaction using anti-V5 antibody. The degradation efficiency of TUSC3-V5 protein was determined by Western blot analysis with anti-ubiquitin antibody. **e,f** *In vivo* ubiquitination assay showing reduced p53 **(e)** or IRE1α **(f)** protein degradation upon TUSC3 overexpression. pcDNA-Ubiquitin-HA, pCMV6-HRD1-Flag and pcDNA-TUSC3-V5-His were co-transfected with pcDNA-P53-GFP **(e)** or pcDNA-IRE1α-GFP **(f)** in HEK293 cells. After 24 h, the cells were incubated with MG132 (8 uM) for 6 h. Subsequently, immunoprecipitation reactions were performed using anti-HA antibody. The ubiquitinated proteins were analyzed by Western blot analyses using indicated antibodies. **g,h,** Restored expression of HRD1 substrates by suppressing HRD1 expression in TUSC3 deficient cells. siHRD1 siRNAs were transfected into A549 TUSC3KD (sh#3A1) cells for 24 h, and the cells were incubated with 1.0 uM of TG for additional 3 h **(g)** or 16 h **(h)**. The restored expression of p53 **(g)** or IRE1a **(h)** was determined by Western blot analysis. **i,** Rescued p53 protein expression by TUSC3 or CCSS mutant. H460 or TUSC3 KO cell was transfected by pcDNA-TUSC3-V5-His or pcDNA-TUSC3-CCSS-V5-His mutant. After 24 h, the cells were treated by 5.0 ug/ml of TM for 6 h and subsequently prepared for Western blot analysis using p53 antibody. **j,k,** Summary tables of IHC scores for p53 **(j)** or IRE1α **(k)** with TUSC3 protein in primary and metastasized lung cancer patient samples. *P*-value was calculated by Chi square analysis.

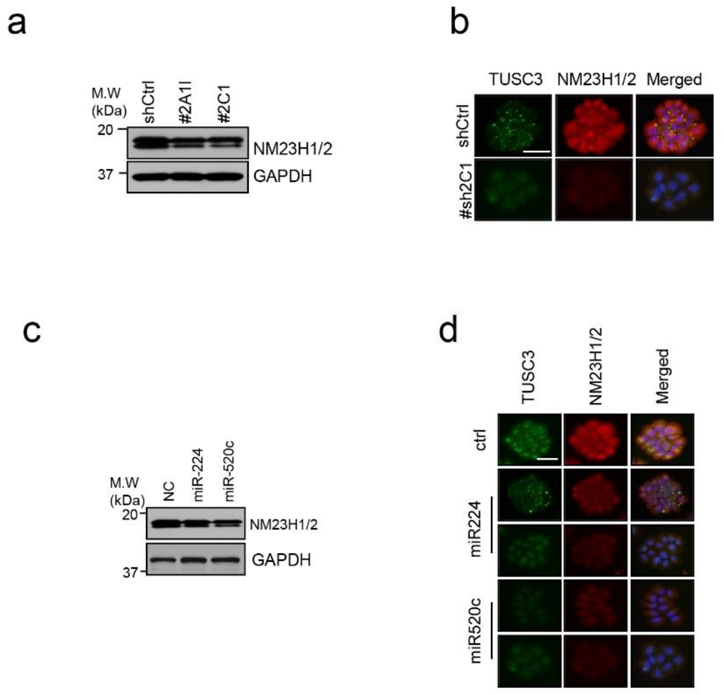

Supplementary Figure 9 The suppression of NM23H1/2 proteins in TUSC3 deficient cells. **a,b,** Reduced NM23H1/2 expression in TUSC3 deficient cells. A549 TUSC3KD (sh#2A1 and #2C1) cells were harvested for Western blot **(a)** or Immunofluorescence **(b)** analysis. **c,d,** miR-224 and -520c-dependent NM23H1/2 suppression. H460 cells were transfected with pre-miR-224 or pre-miR-520c for 48 h. Subsequently the cells were harvested and subject to Western blot analysis **(c)** or Immunofluorescence **(d)**. The scale bars on the images of **(b)** and **(d)** indicate 100 µm.

Fig. 1e, left panel

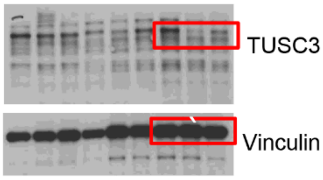

Fig. 1e, right panel

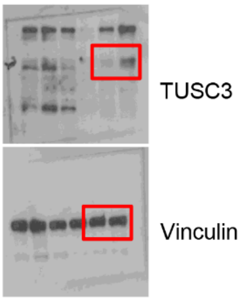

Fig. 2e, left panel

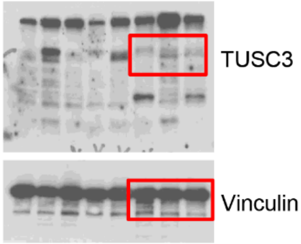

Fig. 2e, right panel

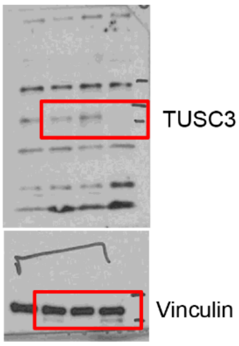

Fig. 3b

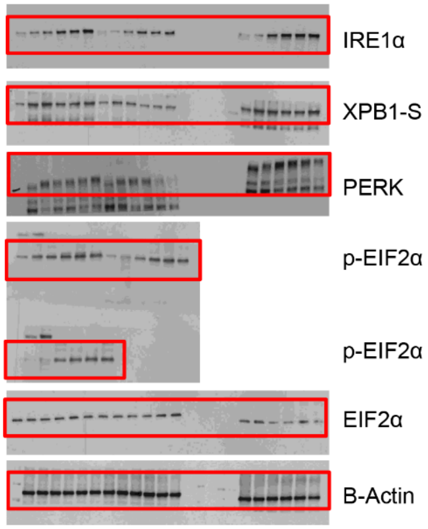

Fig. 3c

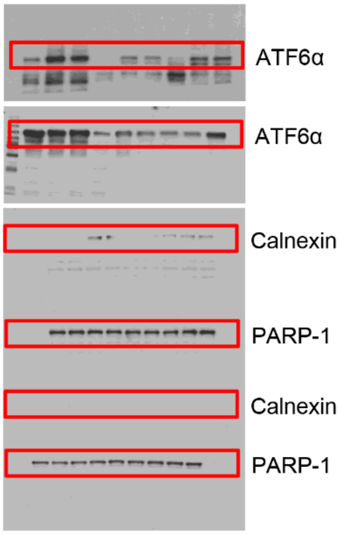

Fig. 3d

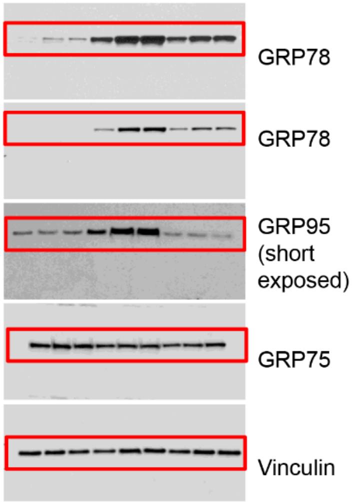

Fig. 3f

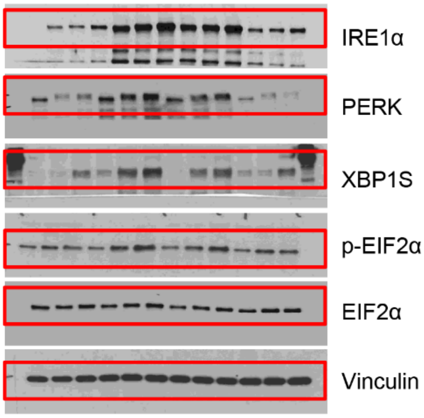

Fig. 3g

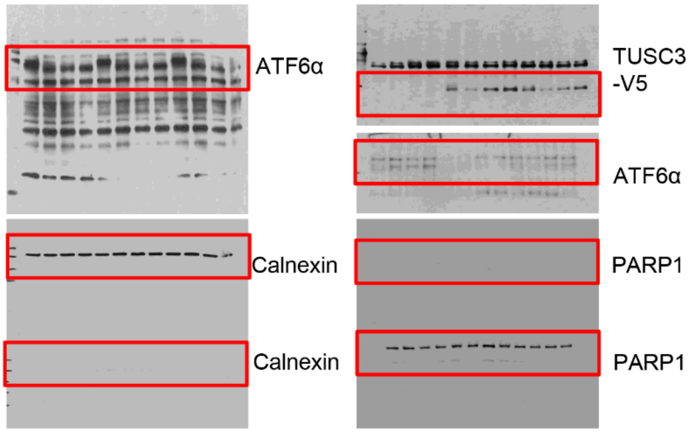

Fig. 4a

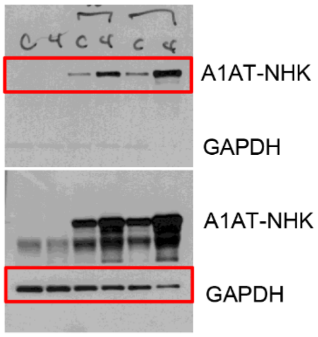

Fig. 4b

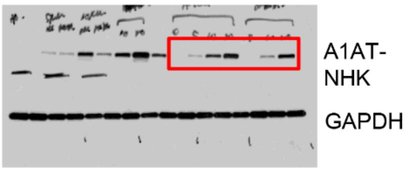

Fig. 4c

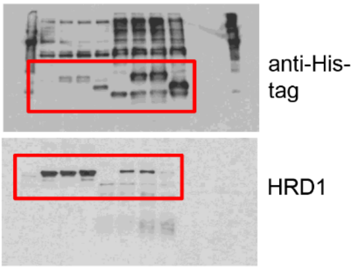

Fig. 4d

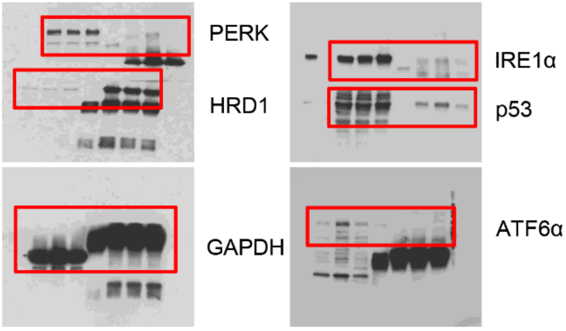

Fig. 4e

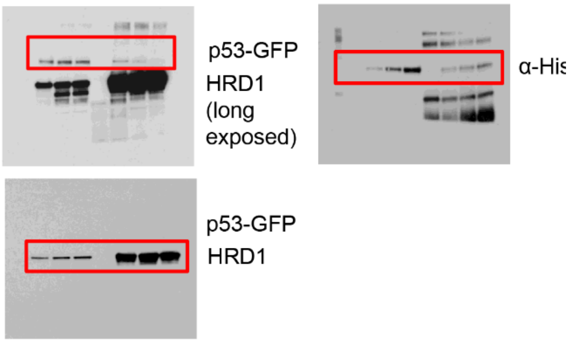

Fig. 4f

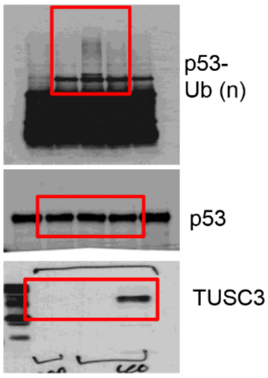

Fig. 4g

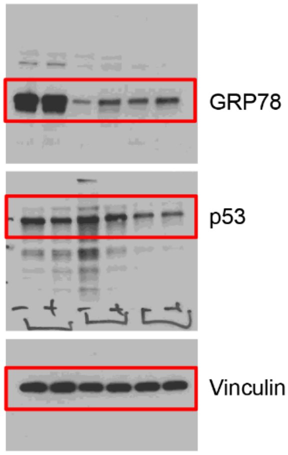

Fig. 4h

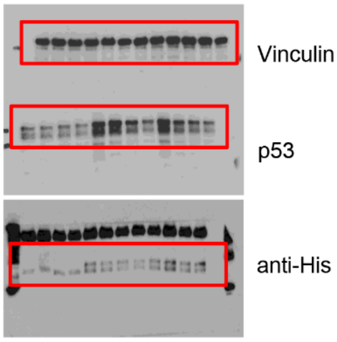

Fig. 5a

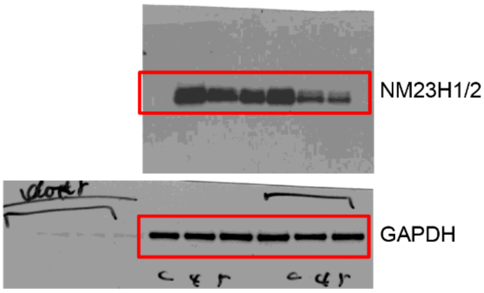

Fig. 5b

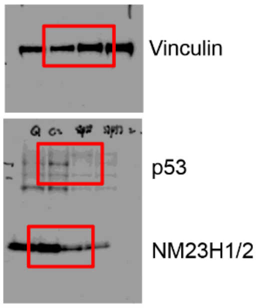

Fig. 5c

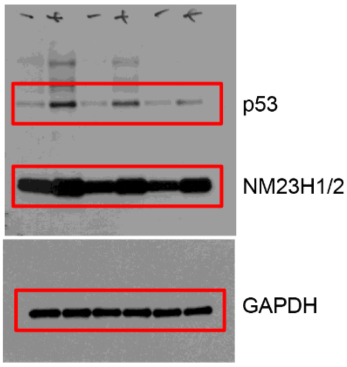

Fig. 5d

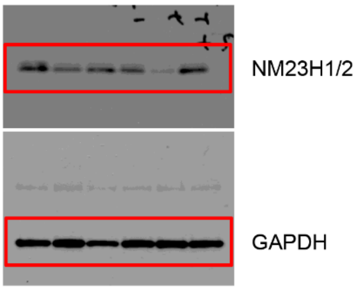

Fig. S5a

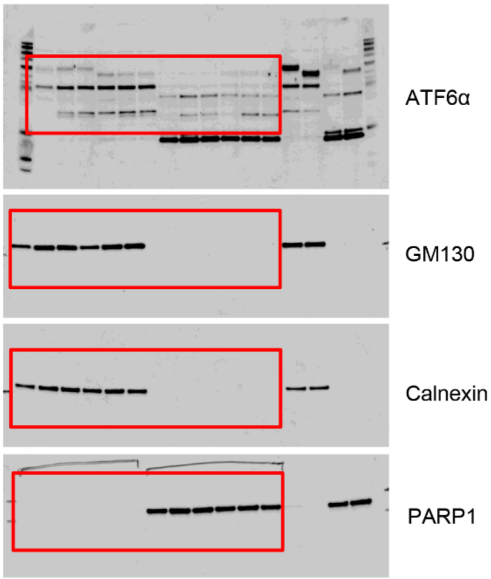

Fig. 5e

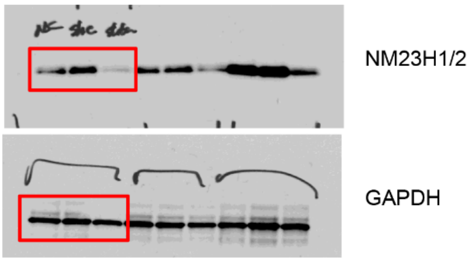

Fig. S6a

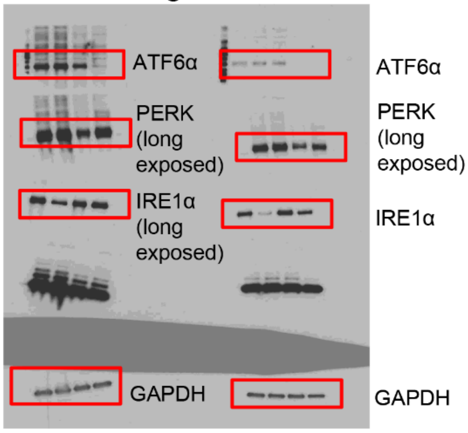

Fig. S5f

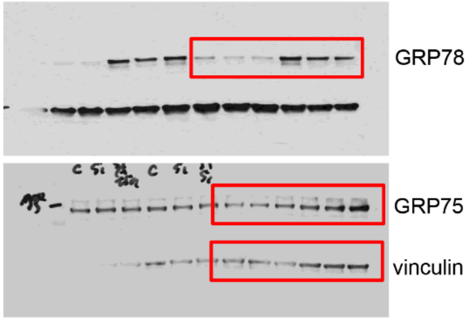

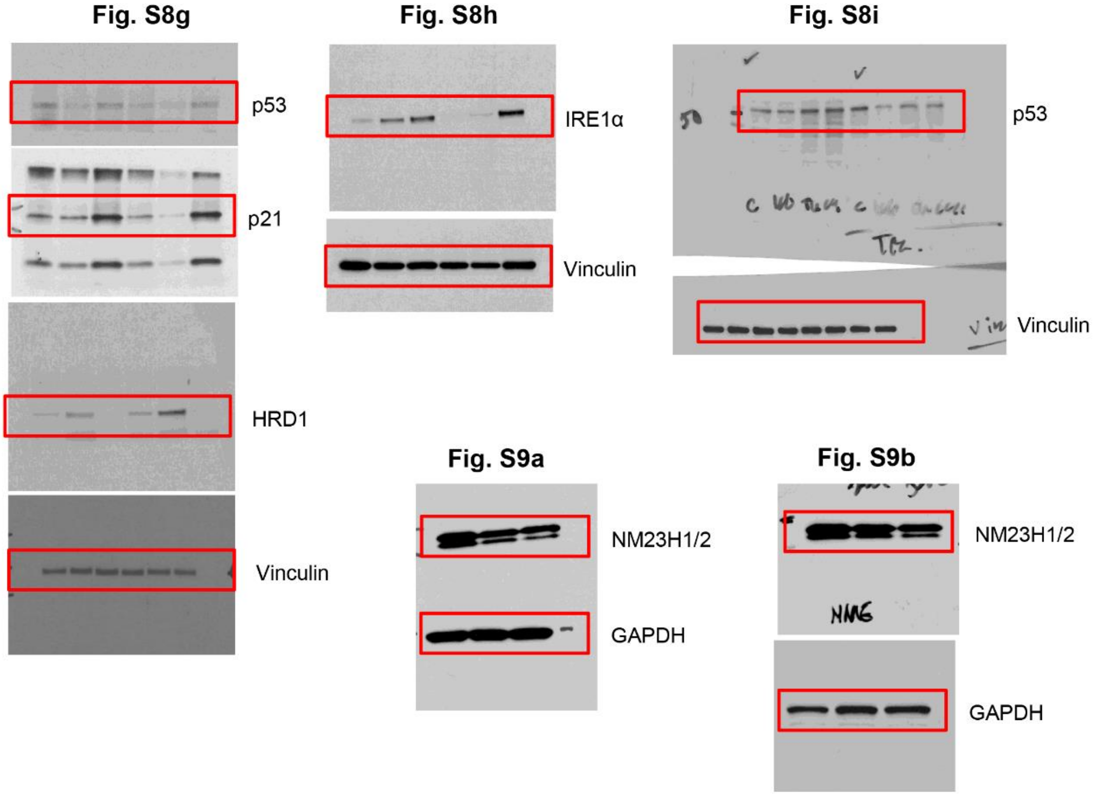

Supplementary Figure 10 Uncropped scan images for Western blot analyses used in the current study.

Supplementary Dataset 1-4 are provided in Excel Files

Supplementary Table 1. The information about the primers, shRNAs and sgRNAs used in the current study

Name	Sequence (5'-3')
TUSC3 full length cDNA	Forward - ATGGGGGCCCGGGGCGCTCCTTCACG;<br>Reverse - CTCAAAGTCCAGATCACTATAAGG
3'UTR of TUSC3-1	Forward - CGGGAAGATGTGATTTGGACCATGG;

	Reverse - CGGCATAATTACATTGTCATTTATTTC
TUSC3 silent mutant -1	Forward - TTTCAACAACTCAACATGAAC; Reverse - CAGAGTTCATGTTGAGTTGTTG
TUSC3 silent mutant -2	Forward - TGACCGAACCGATGTTCATATTC; Reverse - AACATCGGTTCGGTCAGCAATCC
TUSC3 silent mutant -3	Forward - CACACAATGGACAGGTCAGCTAC; Reverse - ATGAATGTAGCTGACCTGTCC
A1AT-NHK	Forward - GGCCGCAACATGCCGTCTTCTG; Reverse - TTAAACGGGCCCTCTAGATTAC
A1AT-NHK-RT-PCR	Forward - ATGGGCCGCAACATGCCGTCTTCTG; Reverse - TGGCCTCTTCGGTGTCCCCGAAGTTGAC
A1AT-NHK-RT-PCR	Forward - CACAAGCTGGAGTACAACTACAACAG; Reverse - TTAAACGGGCCCTCTAGATTAC
TUSC3 3'UTR-1 for miR-520	Forward - TGTGATTTGGACCATGAAAAACTCTATAACC; Reverse - AGAGTTTTTCATGGTCCAAATCACATC
TUSC3 3'UTR-1 for miR-224	Forward - GTGAATGTTTACCATGAAGATAAATACTATTTTG; Reverse - TGAATGAATTCAAAATAGTATTTATCTTCATGG
NM23-H1 full length cDNA	Fowrad - ATGGTGCTACTGTCTACTTT; Reverse - TCATTCATAGATCCAGTTCTGAG
XBP1 primers	Forward - CCTGGTTGCTGAAGAGGAGG; Reverse - CCATGGGGAGATGTTCTGGAG
TUSC3 sgRNAs	1. CGCTCAATCTTCCGAATGAA; 2. GCCCTGTTAGTGTCGCTTGT; 3. CATTCGGAAGATTGAGCGTC
Genotyping primers-A for TUSC3 KO	Forward primer - TTTTACTGGGCTTCCCCTTT; Reverse primer - ACCTGCACACAGAACACTGC
Genotyping primers-B for TUSC3 KO	Forward primer - GGGTGGCATGTTTCTGAGTT; Reverse primer - TGCCAAGTACAGAGGGACAG
HRD1 sgRNAs	1. CAAGTATGTGCTGCACTCCG; 2. GTATGCCATCCTGATGACGA; 3. GGCCAGGGCAATGTTCCGCA
Genotyping primers-A for HRD1 KO	Forward primer - CCTATGCTGAGCTCCTGACC; Reverse primer - AGGCCATGTACAGCAGAACC

Genotyping primers-B for HRD1 KO	Forward primer - CCAACATTGCAGCTTTTCCT; Reverse primer - TCCTCCACATCCTCATCACA
TUSC3 shRNAs	1. CAGCAGCTCAACATGAACT; 2. CTGACAGAACGGATGTTCA ; 3. GACAAGTGAGCTACATTCA
ATF6 shRNAs	1. CACTGATGAGCTGCAATTGGAAGCAGCAA; 2. TTGTCAGTCTCGCAAGAAGAAGAAAGAAT
TUSC3 siRNAs	Purchased from Dhamarcon Company (L-019027-00-0005)
TP53 siRNAs	Purchased from Dhamarcon Company (L-003329-00-0005)
HRD1 siRNAs	Purchased from Dhamarcon Company (L-003329-00-0005)
ATF6 siRNAs	Purchased from Dhamarcon Company (L-009917-00-0005)
PERK siRNAs	Purchased from Dhamarcon Company (L-004883-00-0005)
IRE1 siRNAs	Purchased from Dhamarcon Company (L-004951-02-0005)
BIP siRNAs	Purchased from Dhamarcon Company (L-008198-00-0005)

Supplementary Table 2. Probes or amplicon information for qRT-PCR used in the current study

Gene Name	Assay ID	Primers for RT-PCR amplicon
**TUSC3**	**Hs00954406_m1***	**Forward- atggaatggagttccagacg; Reverse- gtttcgaggtggtgccttta**
**HRD1/SYVN1**	**Hs00381211_m1**	**For - ctgaggaccgtgtggacttt; Reverse - gatgcccaggaggaacataa**
**NME1**	**Hs00897129_m1**	
**NME2**	**Hs00267363_m1**	
**TP53**	**Hs01034249_m1**	
**hsa-miR-224**	**2099**	
**hsa-miR-520c-3p**	**2400**	
**hsa-miR-373**	**561**	
**hsa-miR-373***	**562**	

References

1 Herroon, M. K., Rajagurubandara, E., Diedrich, J. D., Heath, E. I. & Podgorski, I. Adipocyte-activated oxidative and ER stress pathways promote tumor survival in bone via upregulation of Heme Oxygenase 1 and Survivin. *Scientific reports* **8**, 40, doi:10.1038/s41598-017-17800-5 (2018).

2 Nuovo, G. False-positive results in diagnostic immunohistochemistry are related to horseradish peroxidase conjugates in commercially available assays. *Annals of diagnostic pathology* **25**, 54-59, doi:10.1016/j.anndiagpath.2016.09.010 (2016).
