# Supplementary material for: miRNA-mediated TUSC3 deficiency enhances UPR and ERAD to promote metastatic potential of NSCLC
Source: Nat Commun. 2018 Nov 30;9:5110. doi: 10.1038/s41467-018-07561-8 (PMC6269493; doi:10.1038/s41467-018-07561-8)
Supplement: Supplementary file 7 — Description of Additional Supplementary Files [file 41467_2018_7561_MOESM7_ESM.docx]

**Title:** Supplementary Dataset 1.
**Description:** The lists of genes in PCR array

**Title:** Supplementary Dataset 2.
**Description:** The lists of genes associated with metastasis in TUSC3KO with GSEA

**Title:** Supplementary Dataset 3.
**Description:** The lists of genes regarding unfolded protein response in TUSC3KO cells.

**Title:** Supplementary Dataset 4.
**Description:** The lists of genes associated p53 activation in TUSC3KO cells.
